# TangBi Formula for Painful Diabetic Distal Symmetric Polyneuropathy: A Multicenter, Randomized, Double‐Blind, Placebo‐Controlled and Parallel‐Group Trial

**DOI:** 10.1111/1753-0407.70045

**Published:** 2025-01-05

**Authors:** Xuefei Zhao, Xuedong An, Yashan Cui, Liu Dong, Zhaohui Fang, Zhonghua Zheng, Xinhe Zuo, Huailin Gao, Tianshu Gao, Qing Ni, Fengmei Lian, Xiaolin Tong

**Affiliations:** ^1^ Guang'anmen Hospital China Academy of Chinese Medical Sciences Beijing China; ^2^ Xiyuan Hospital China Academy of Chinese Medical Sciences Beijing China; ^3^ The First Affiliated Hospital of Anhui University of Traditional Chinese Medicine Hefei Anhui Province China; ^4^ Zhengzhou Hospital of Traditional Chinese Medicine Zhengzhou China; ^5^ Hubei Provincial Hospital of Traditional Chinese Medicine Wuhan Hubei Province China; ^6^ Hebei Yiling Hospital Shijiazhuang Hebei Province China; ^7^ The First Affiliated Hospital of Liaoning University of Traditional Chinese Medicine Shenyang Liaoning Province China

**Keywords:** diabetes, diabetic distal symmetric polyneuropathy, herbal therapy, TCM

## Abstract

**Objective:**

To determine whether the use of herbal medicines combined with conventional treatment is more effective than conventional medication alone in improving clinical symptoms in patients with diabetic distal symmetric polyneuropathy (DSPN).

**Methods:**

This multicenter, placebo‐controlled, double‐blind, randomized controlled clinical trial recruited patients from 6 clinical centers in mainland China. A total of 188 patients were randomly assigned in a 1:1 ratio to the treatment group (Tangbi Formula plus methylcobalamin) and the control group (placebo plus methylcobalamin). Subjects were reassessed after the 24‐week intervention. The primary outcomes were differences in changes in clinical signs and symptoms and changes in the Michigan Diabetic Neuropathy Score (MDNS) between the two groups before and after treatment. Secondary outcomes were changes in nerve conduction velocity (NCV) and single clinical signs and symptoms as measured by the visual‐analogue scale (VAS) and Toronto clinical scoring system (TCSS).

**Results:**

Compared with the placebo group, after 24 weeks of treatment, the MDNS score of TangBi Formula group was significantly reduced (*p* < 0.001). There were no significant changes in NCV results in either group before or after treatment. Compared with baseline, the difference in the change value of VAS score between the two groups after treatment was statistically significant (*p* = 0.031). A statistically significant difference in the change value of TCSS after treatment compared to baseline was found between the two groups (*p* = 0.033 at 12 weeks and *p* = 0.030 at 24 weeks). No severe adverse events due to study participation or study intervention were reported.

**Conclusions and Relevance:**

This trial demonstrated that combining Tangbi Formula with basal therapy can be safer and more effective in improving the symptoms of DSPN patients.

## Graphical abstract

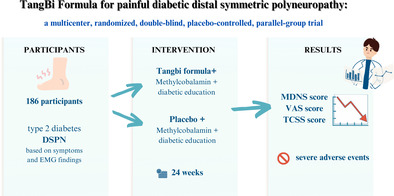

**Summary**

- The results of this multicenter, placebo‐controlled, double‐blind, randomized controlled clinical trial suggest that combining TangBi Formula with routine treatments may provide a safer and more effective way to alleviate the symptoms of DSPN.

**Abbreviations**
AE: adverse events
CRF: case report form
DPN: diabetic peripheral neuropathy
DSPN: diabetic distal symmetric polyneuropathy
FAS: full analysis set
HGWD: Huangqi Guizhi Wuwu Decoction
MDNS: Michigan Diabetic Neuropathy Score
MNCV: motor nerve conduction velocity
NCV: nerve conduction velocity
PPS: per protocol set
SAE: serious adverse events
SD: standard deviation
SNCV: sensory nerve conduction velocity
SS: safety analysis set
T1DM: Type 1 diabetes mellitus
T2DM: Type 2 diabetes mellitus
TCM: traditional Chinese medicine
TCSS: Toronto clinical scoring system
VAS: visual‐analogue scale

## 1 Introduction

Diabetes is a global public health crisis affecting 537 million people worldwide, with the number of patients increasing annually and trending toward younger age groups [1]. A Chinese nationally representative cross‐sectional study estimated the overall prevalence of diabetes in China to be 12.4%, while only 32.9% of patients were receiving treatment, and 50.1% of them were adequately controlled [2]. The damage caused by diabetes is systemic, which has a profound impact on quality of life. The global epidemic of diabetes has led to a corresponding epidemic of its complications. The most common complication is diabetic peripheral neuropathy (DPN), particularly distal symmetric polyneuropathy, which ultimately occurs symptomatically or insidiously in 45% of patients with Type 2 Diabetes Mellitus (T2DM) and 54% of patients with Type 1 Diabetes Mellitus (T1DM) [3]. It is defined as a progressive bilateral symmetrical sensory dysfunction and chronic neuropathic pain beginning in the distal lower extremities after other etiologies have been ruled out, with the following symptoms including burning pain, tingling sensation, abnormal sensation, sensory hypersensitivity and deep pain [4]. DPN is an insidious disease with few or no early symptoms, but the prognosis is quite dismal and has a profound impact on quality of life. Currently, much emphasis is generally placed on taking preventive measures, education, and early screening, while the vast majority of symptomatic medications have minimal efficacy, with less than one in seven patients with DPN pain achieving adequate pain relief [5]. However, the impact of DPN is enormous. It is associated with reduced quality of life, falls and epidermal injuries leading to infection and amputation, as well as psychological changes and comorbidities, including greater anxiety and depression and sleep disturbances [6].

Current directions in the treatment of DPN are focused on improving glycaemic control (including lifestyle interventions and glucose‐lowering medications), improving other indicators of metabolic syndrome and drug‐based neuropathic pain management to slow progression and improve symptoms [3]. However, glycaemic control is only effective in patients with type 1 diabetes, whereas neuropathy is far less likely to be reduced in patients with type 2 diabetes [7]. The anticonvulsants pregabalin and gabapentin both have an A level of evidence for effectiveness in the EFNS and AAN guidelines, but their evidences were weaker in the high‐quality studies and patients do not adhere to their use over time [8, 9]. Opioid and Atypical Opioid Analgesics, like tramadol and oxycodone, have no evidence of long‐term efficacy and are addictive, although they may be effective in reducing pain in patients with DPN in the short term. Amitriptyline is a tricyclic antidepressant that is a first‐line medication recommended by many guidelines for its low price, but few clinical studies have been reported on its efficacy, pain relief, and impact on life satisfaction [10]. Because the mechanisms of DSPN are still incompletely understood, there remains a lack of pathogenesis‐based medications to significantly alter the natural history of the disease. Additional non‐pharmacological therapies such as psychological support, acupuncture, physiotherapy and low‐frequency electrical spinal cord stimulation or muscle stimulation are also used in clinical practice [11]. It is important to note that pain management approaches vary from patient to patient and some sufferers show limited response to current treatments, so there is still a need to develop new interventions.

Perhaps due to the difficulty of resolving patients' problems with existing conventional therapies, a number of DSPN patients in China seek help from traditional Chinese medicine (TCM). In recent years, TCM combined with western medicine for the treatment of DPN has been proved to be effective in relieving patients' clinical signs and symptoms and significantly improving NCV [12]. A clinical study showed that Xiaoketongbi Formula was effective in treating patients with DPN, with improvements in pain comparable to Pregabalin [13]. TangBi Formula is based on the classic TCM formula of A Huangqi Guizhi Wuwu Decoction (HGWD), which consists of granules made from the following five herbs, known as Astragalus membranaceus (Huangqi), cassia twig (Guizhi), Ligusticum chuanxiong Hort (Chuanxiong), Radix Paeoniae Alba (Baishao), and Caulis Spatholobi (Jixueteng). In addition, a retrospective study showed that modified HGWD was effective in treating moderately severe painful DPNs for which conventional medications failed, particularly for three common symptoms: limb pain, limb numbness, and insomnia [14]. The results of our preclinical study showed that modified HGWD could delay the decline in nerve conduction velocity, the progression of diabetic peripheral neuropathy, make patients more sensitive to temperature and pain sensation, and improve haemorheology‐related indices [15, 16]. HGWD has also been found to be helpful in neurological damage caused by chemotherapeutic agents, such as Oxaliplatin and Paclitaxel, by inhibiting inflammatory and oxidative responses in the peripheral nervous system through the TLR4/NF‐κB and PI3K/Akt‐Nrf2 pathways [17, 18]. It has also been investigated the role of HGWD in ameliorating intestinal barrier damage, decreasing serum LPS and related inflammatory factor levels in the colon, serum, and DRG, and thereby improving pain sensitivity from a gut‐peripheral nerve axis perspective [19]. We optimized HGWD through our long‐term practical experience in clinical practice to form TangBi Formula, which has been patented (Patent No. ZL 2021 11403484.0). Therefore, TangBi Formula may have a better effect on diabetes‐induced peripheral neuropathy, and we have observed this efficacy in clinical practice, although evidence‐based is still lacking.

The aim of this clinical trial is to assess whether combining herbal medicine with conventional management methods can be more effective in slowing down or even reversing the progression of the disease and provide better pain management.

## 2 Materials and Methods

### 2.1 Study Design

This was a multicenter, randomized, double‐blind, placebo‐controlled, parallel‐group clinical trial carried out from June 2017 to January 2020 at 6 clinical centers in mainland China, with 24 weeks of intervention. The study was registered with ClinicalTrials.gov, NCT03010241. The entire study was approved by the local ethical committee of Guang'anmen Hospital, Chinese Academy of Chinese Medical Sciences (Ethics Approval No. 2016‐093‐KY‐01). The methodology is detailed in the Supplement and previously published study protocol.

This was a multicenter, randomized, double‐blind, placebo‐controlled, parallel‐group clinical trial conducted from June 2017 to January 2020 at six clinical centers in mainland China, with a 24‐week intervention period. The study was registered with ClinicalTrials.gov, NCT03010241. The study was approved by the local ethics committee of Guang'anmen Hospital, Chinese Academy of Chinese Medical Sciences (Ethics Approval No. 2016‐093‐KY‐01). The methodology is outlined in detail in the supplement and in the previously published study protocol [20].

### 2.2 Participants

Recruitment for this study was conducted at six clinical centers, including Guang'anmen Hospital of the China Academy of Chinese Medical Sciences, the First Affiliated Hospital of Liaoning University of Traditional Chinese Medicine, the First Affiliated Hospital of Anhui University of Traditional Chinese Medicine, Hubei Provincial Hospital of Traditional Chinese Medicine, Zhengzhou Hospital of Traditional Chinese Medicine, and Hebei Yiling Hospital (Table S1). Individuals were eligible to participate in the study if they had physician‐diagnosed diabetic DSPN, with numbness of the limb extremities; spontaneous pain (e.g., burning pain, tingling, dull pain); and paresthesia, usually distributed like gloves or socks, starting from the lower limbs, symmetrically; foot pain sense and diminished temperature sensation, vibration decreased or disappeared, but motor function basically intact. The included patients had decreased NCV on electromyography and were between 40 and 79 years of age. Key exclusion criteria were recent use of antioxidants, such as vitamin E or vitamin C; acute infection; liver or kidney dysfunction; acute complications of diabetes; severe cardiovascular and cerebrovascular disease; and neuropathy caused by long‐term alcohol consumption or other factors.

### 2.3 Sample Size

Based on the preliminary study of TangBi prescription data, the difference in MDNS scores between the placebo group was 3.45, and 4.13 in TangBi prescription, with a standard deviation of 1.33. To determine the appropriate sample size, a hypothesis test formula for measurement data was used: *N* = 2 × [(Zα + Zβ) × δ/d]2. In this formula, N represents the sample size, δ is the estimated standard deviation, d is the mean difference between the two groups' continuous variables, and Zα and Zβ are the corresponding standard normal differences. Assuming a significance level (α) of 0.05 and a (1 − β) of 0.9 out 0, the quantile Zα was found to be 1.64485 and Zβ was 0.84162, which were then entered into the formula. Considering the 20% dropout and withdrawal rates, it was estimated that 78 subjects would be needed in each group, and a total of 188 participants would be included in the study when fully considered. To minimize bias caused by dropouts, intention‐to‐treat analysis will be employed.

### 2.4 Randomization and Blinding

A total of 188 patients were included according to the sample size calculations. All eligible and consenting participants underwent baseline assessment and clinical data collection, before being randomly assigned in a 1:1 ratio to either the control or intervention group. To eliminate potential central effects and minimize bias, this multicenter study employed centralized randomization. Specific randomization lists were generated by computer and safeguarded from researchers by senior data managers who were not involved in the study. The lists were kept confidential and not accessible to researchers. The distribution lists are safeguarded by password‐protected files and are maintained by independent non‐research personnel. Once production was complete, the study drug was dispensed into numbered vials by a designated pharmacist, based on a randomized list prepared by the trial statistician. The treatment course lasted 6 months. All trial participants, including patients, investigators, project manager, data management team, clinical research assistants, and statisticians, were blinded to group assignment. The analysis of results was conducted by analysts who were unaware of the group assignment. These were achieved through a centralized randomization system, the code for which was in the hands of an independent allocator and was disclosed at the end of the treatment period and analysis.

### 2.5 Intervention

Patients received either Tangbi Formula (6 g/bag twice a day) or a placebo capsule twice a day for 24‐weeks. Each participant received conventional treatment consisting of oral Methylcobalamin (500 mg tablets three times a day), diabetic education, diet, rational control of blood glucose, and other oral medications. In principle, the glucose‐lowering drug species and dosage used should be kept invariable, and steady blood glucose should be controlled as much as possible throughout the course of the trial. If fasting blood glucose fluctuates more than 20%, a diabetes specialist should be consulted to find and exclude the triggering factors soon, adjust the drug dose, and monitor the blood glucose changes so that it can be reduced to a stable level within 4 weeks. The adjusted dosage should be recorded in detail in the Combined Medication Chart.

### 2.6 Assessments

All participants underwent assessment through planned clinic visits at seven time points (at baseline and at 4, 8 12, 16 and 24 weeks after baseline). Three scales, MDNS, VAS and TCSS were used to evaluate the patients after 12 and 24 weeks of treatment, respectively and the changes of scores before and after dosing were compared between the two groups. Changes in NCV were used to quantitatively assess efficacy and were tested at week 0 and week 24 to compare with each other. At each time point, blood pressure and fasting blood glucose were assessed. Other secondary evaluators included blood pressure and fasting blood glucose assessed at each time point, as well as glycosylated hemoglobin, and hematological lipids assessed every 3 months. All assessments were conducted by a trained research assistant who was blinded to the participants' randomization status. Furthermore, there were monthly recorded vital signs: such as temperature, blood pressure, respiration, heart rate, and so on, and every 3 months for routine blood, urine, and stool test, as well as electrocardiograms, liver function tests (alanine aminotransferase, aspartate aminotransferase, γ‐glutamyl transpeptidase, alkaline phosphatase, and total bilirubin), and renal function tests (blood urea nitrogen and serum creatine) for safety assessment. In this study, paper research charts and case report form (CRF) were used to record and preserve the collected data.

### 2.7 Statistical Analysis

All subjects who were randomized and received at least one treatment were enrolled in the Full Analysis Set (FAS). Criteria for eligibility to enter the Per Protocol Set (PPS) were all cases that met the study protocol, were compliant, used 80%–120% of the study medication, completed the CRF with the required fill‐in content, had measurable primary variables, no missing baseline variables, and no major violations of the trial protocol. The Safety Analysis Set (SS) contains all subjects who received at least one treatment after randomization. We listed the enrolled cases and completed cases and reasons for shedding, described the number of cases in each group, and a list of those who did not enter the PPS. A two‐sided *p* < 0.05 was considered statistically significant. All analyses were performed using the SAS9.3 software.

#### 2.7.1 Baseline Equalization

In order to examine the baseline equilibrium, the baseline data were processed as follows: quantitative indicators of the two groups were listed as the number of cases, mean, standard deviation, median, maximum, and minimum values and thus compared with the *t*‐test or Wilcoxon's rank‐sum test; and qualitative indicators were listed as the frequency and percentage and compared with the chi‐square test and Fisher's exact test.

#### 2.7.2 Effectiveness Analysis

After treatment, to depict the primary outcome MDNS and its change relative to baseline, the number of cases, mean, standard deviation, median, minimum, and maximum were calculated for each group, and the paired *t*‐test or the Wilcoxon signed‐rank test was used for within‐group comparisons, while the *t*‐test or the Wilcoxon rank‐sum test was used to make an intergroup comparison of the two groups for the change relative to baseline after treatment. Similar methodology was also applied to describe secondary efficacy indicators which are NCV, single symptom and change relative to baseline. We also used paired *t*‐tests or Wilcoxon signed rank tests for within‐group comparisons of other quantitative forms VAS and TCSS, and between‐group comparisons of change after treatment relative to baseline between the two groups using *t*‐tests or Wilcoxon rank sum tests. Statistical analyses were also performed in accordance with the methods described above for other monitoring indicators, including post‐treatment fasting glucose, glycosylated hemoglobin, blood pressure, lipids, and their changes relative to baseline.

#### 2.7.3 Safety Analysis

The incidence rates of adverse events (AEs) and serious adverse events (SAEs) were calculated. SS were performed on the safety set.

## 3 Results

**FIGURE 1:**
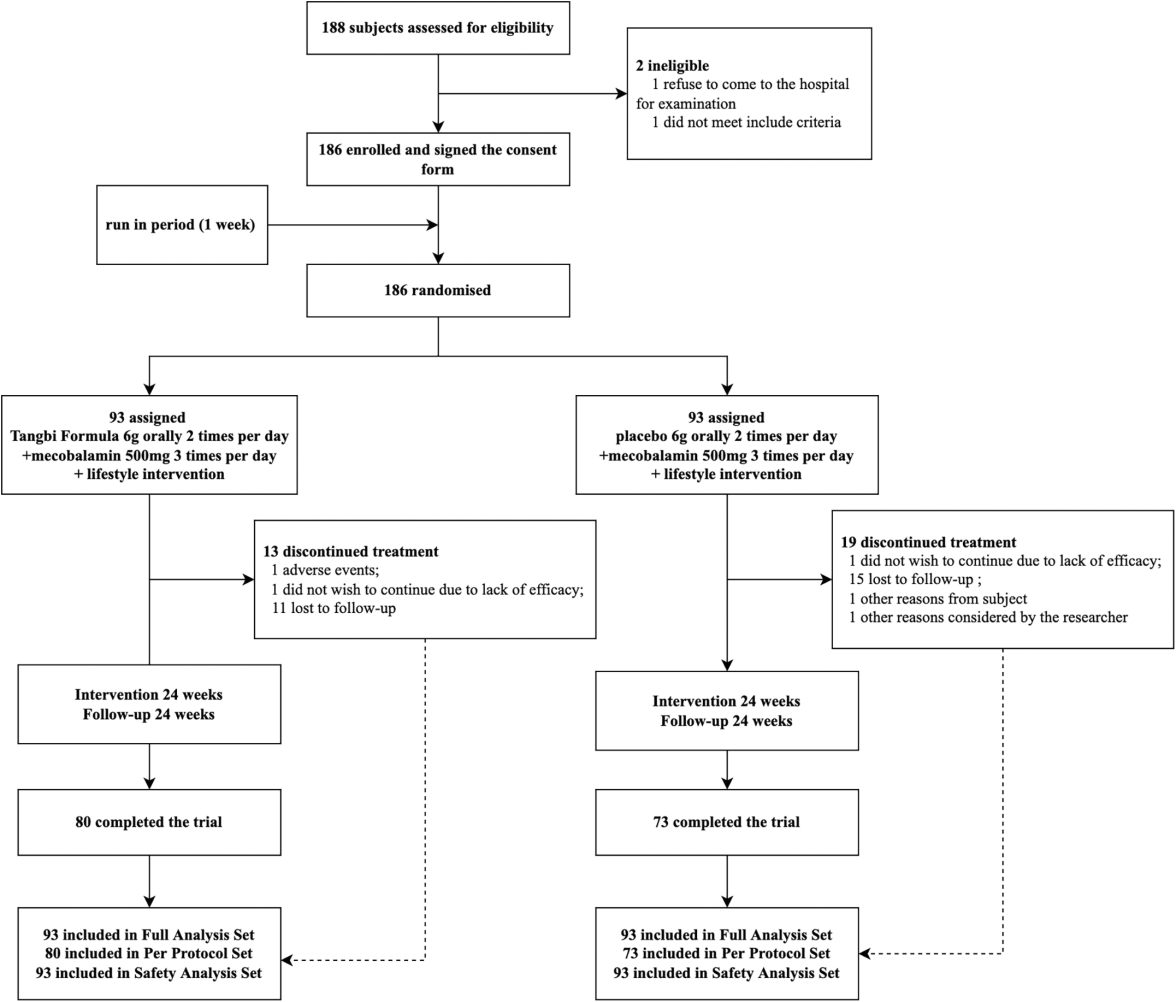
Participant screening flowchart.

**FIGURE 2:**
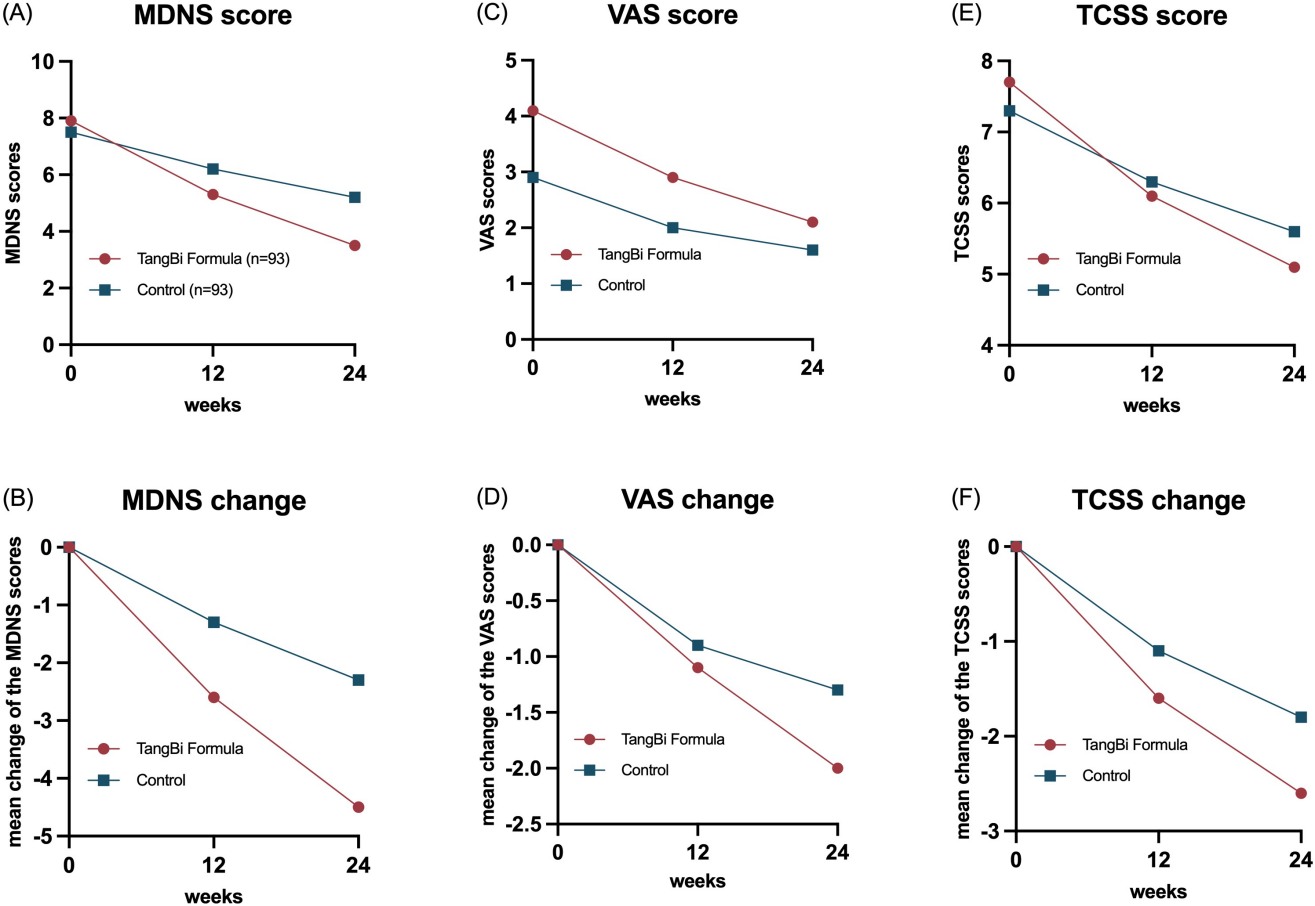
Analysis of changes in outcomes: Mean MDNS scores in both groups over 24 weeks (A); Mean change in MDNS scores in both groups over 24 weeks (B); Mean VAS scores in both groups over 24 weeks (C); Mean change in VAS scores in both groups over 24 weeks (D); Mean TCSS scores in both groups over 24 weeks (E); Mean change in TCSS scores in both groups over 24 weeks (F).

Between June 2017 and January 2020, 188 DSPN patients were screened for the trial. The 186 individuals who met the inclusion criteria were randomized after a 1‐week run‐in period, and 153 of them completed the trial (82.3% completion rate), with 80 in the intervention arm and 73 in the control arm (Figure 1, Further baseline characteristics of the PPS population were recorded in Table S2). The mean (SD) age of patients was 57.5 (SD 8.10) years; 115 (61.8%) were males. Mean duration of diabetes at enrolment was 10.9 (SD 6.72) years in TangBi Formula group and 11.2 (SD 6.00) in the control group, with Diabetes, hypertension, coronary heart disease and hyperlipidemia being the most common comorbidities reported. There were no significant baseline characteristic differences observed between the Tangbi Forma group and the placebo group that might have influences on the assessment of efficacy (Table 1). The results of the FAS are presented in Figure 2.

**TABLE 1:** Baseline characteristics of study patients. (FAS population).

Characteristic	TangBi Formula (*n* = 93)	Control (*n* = 93)	All participants (*n* = 186)	*p*
Age (year), mean (SD)	57.4 (8.54)	57.6 (7.69)	57.5 (8.10)	0.824
Sex, *n* (%)				0.45
Male	55 (59.1%)	60 (64.5%)	115 (61.8%)	
Female	38 (40.9%)	33 (35.5%)	71 (38.2%)	
Height (cm), mean (SD)	166.3 (8.45)	168.1 (7.83)	167.2 (8.18)	0.191
Weight (kg), mean (SD)	68.62 (11.317)	71.12 (12.223)	69.87 (11.814)	0.173
BMI (kg/m^2^), mean (SD)	24.775 (3.3227)	25.071 (3.3608)	24.923 (3.3361)	0.547
Duration of diabetes (year), mean (SD)	10.9 (6.72)	11.2 (6.00)		0.606
Duration of DSPN (year), mean (SD)	2.453 (2.8132)	2.303 (2.3541)		0.821
Comorbid with other diabetic complications (yes), *n* (%)	28 (30.1%)	20 (21.5%)		0.18
Past DSPN treatment history (yes), *n* (%)	23 (24.7%)	26 (28.0%)		0.618
Past usage of drugs for DSPN (yes), *n* (%)	22 (23.7%)	24 (25.8%)		0.734
Past medical history and the usage of drugs (yes), *n* (%)	82 (88.2%)	85 (91.4%)		0.468
Drug combination during the study period (yes), *n* (%)	64 (68.8%)	63 (67.7%)		0.875
Urine pregnancy test (positive), *n* (%)	0 (0.0%)	0 (0.0%)		
FBG (mmol/L), median (p25–p75)	7.340 (6.230, 9.900)	8.280 (7.000, 9.900)		0.105
HbA1c (%), median (p25–p75)	7.600 (6.800, 8.600)	7.600 (6.800, 9.200)		0.477

### 3.1 Primary Outcome (Changes in MDNS)

The MDNS sensory scale (including sensation, muscle strength and reflexes) was used as the primary outcome to assess small fiber nerve function. A comparison of the primary outcomes revealed a significant difference in MDNS scores between the treatment and control groups at 24 weeks (*p* < 0.001), but not at baseline or 12 weeks (Table 2 shows the results for the FAS group and Table S3 shows the results for the PPS group). Following 12 weeks of treatment, a significant difference was observed in the change in scores from baseline levels between the two groups (*p* = 0.002), which was further pronounced after 24 weeks of treatment (*p* < 0.001). In accordance with the MDNS total score reduction of three points, which was identified as the threshold for efficacy, the response rates between the two groups were 20.41%. There were no statistically significant differences between the treatment and control groups at baseline in any of the specific scoring items of the MDNS. After 24 weeks of intervention, all physical examinations were trending toward normal or mild to varying degrees. At the end of treatment, bilateral ankle dorsiflexor strength (left side *p* = 0.022, right side *p* = 0.005), bilateral Achilles tendon reflexes (left side *p* = 0.001, right side *p* = 0.003), and left biceps reflexes (*p* = 0.004) were significantly improved in the TangBi Formula group compared with the control group (detailed in Table S4).

**TABLE 2:** Analysis of changes in primary outcomes (FAS population), mean (SD).

	TangBi Formula (*n* = 93)	Control (*n* = 93)	*p*
At baseline	7.9 (7.93)	7.5 (8.11)	0.19
12 weeks	5.3 (5.43)	6.2 (6.08)	0.258
12‐week changes	−2.6 (4.10)	−1.3 (3.32)	0.002*
LS mean (95%CI)	−2.53 (−3.04, −2.01)	−1.38 (−1.90, −0.86)	0.002*
LS mean of difference (95%CI)	1.15 (0.42, 1.88)		
24 weeks	3.5 (4.22)	5.2 (4.48)	< 0.001*
24 weeks changes	−4.5 (5.85)	−2.3 (5.33)	< 0.001*
LS mean (95%CI)	−4.39 (−5.05, −3.72)	−2.40 (−3.06, −1.73)	< 0.001*
LS mean of difference (95%CI)	1.99 (1.05, 2.93)		
Percentage of MDNS decreasing by 3 points after 24 weeks	44.90%	24.49%	
Response rate	20.41%		

Abbreviations: LS mean, least square mean; SD, standard deviation.

* *p* < 0.05.

### 3.2 Secondary Outcomes (Changes in NCV)

The motor nerve conduction velocity (MNCV) of the right median nerve and the sensory nerve conduction velocity (SNCV) of the left peroneal nerve, left sural nerve on both sides tended to increase in the TangBi Formula group but decreased in the placebo group. There was no significant difference in the change of conduction velocity of individual nerves between the groups (*p* > 0.05). See Table S5 for details.

### 3.3 Other Outcomes

#### 3.3.1 Changes in VAS

The mean pain VAS scores at baseline, 12 weeks, and 24 weeks were 4.1, 2.9, and 2.1 in the patients in the TangBi Formula group and 2.9, 2.0, and 1.6 in the placebo group, in that order. Although there was a statistically significant difference between the two groups at baseline, VAS scores in both groups decreased compared with baseline levels (*p* < 0.001) and the between‐group difference was statistically significant (*p* = 0.046) after 12 weeks of treatment. After 24 weeks of treatment, there was a statistically significant difference between the values of change in VAS in the two groups compared to baseline (*p* = 0.031). At the end of 24 weeks of treatment 28 patients in the intervention group achieved pain relief, defined as a VAS score of 3 cm or less. (Table 3 shows the results for the FAS set and Table S6 for the PPS set).

**TABLE 3:** Analysis of changes in VAS (FAS population), mean (SD).

	TangBi Formula (*n* = 74)	Control (*n* = 71)	*p*
At baseline	4.1 (3.38)	2.9 (3.21)	0.026*
12 weeks	2.9 (2.82)	2.0 (2.53)	0.046*
12‐week changes	−1.1 (1.31)	−0.9 (1.26)	0.181
24 weeks	2.1 (2.40)	1.6 (2.23)	0.162
24 weeks changes	−2.0 (1.97)	−1.3 (1.75)	0.031*

* *p* < 0.05.

#### 3.3.2 Changes in TCSS

The mean values of TCSS in the baseline TangBi Formula group and the control group were 7.7 and 7.3, respectively, and there was no statistical difference between the two groups (*p* = 0.419). It was clear that after 12 and 24 weeks of treatment, the mean values of TCSS were gradually decreasing in both groups. More specifically the TangBi Formula group showed a decrease in mean TCSS values from baseline of 1.6 at 12 weeks and 2.6 at 24 weeks. In the control group, the mean TCSS values from baseline decreased by 1.1 at 12 weeks and 1.8 at 24 weeks, and there was a statistically significant difference in the values of the changes between the two groups. (Table 4 shows the results for the FAS set and Table S7 for the PPS set).

**TABLE 4:** Analysis of changes in TCSS (FAS population), mean (SD).

	TangBi Formula (*n* = 93)	Control (*n* = 93)	*p*
At baseline	7.7 (2.76)	7.3 (2.48)	0.419
12 weeks	6.1 (2.35)	6.3 (2.68)	0.986
12‐week changes	−1.6 (1.72)	−1.1 (1.36)	0.033*
24 weeks	5.1 (2.24)	5.6 (2.78)	0.651
24 weeks changes	−2.6 (2.11)	−1.8 (1.48)	0.030*

* *p* < 0.05.

### 3.4 Safety

There was no significant difference in medication adherence between the two groups (96.572% vs. 94.995%, *p* = 0.901). No serious adverse events were reported during the treatment period in either group, and the incidence of AEs was not significantly different between the Tangbi Formula group and the control group (6.5% *n* = 6 vs. 6.5% *n* = 6). In the Tangbi Formula group, two subjects developed mild elevations of ALT and AST, two tested for urinary protein, and one had elevated blood acid phosphatase, all of which eventually resolved without dosage adjustments and completed the study according to the treatment plan, except for one patient who ultimately withdrew from the study due to hepatic function abnormalities. Also one case in the placebo group withdrew from the trial due to chronic renal insufficiency.

## 4 Discussion

In this randomized clinical trial targeting Chinese patients with DSPN, TangBi Formula, as an adjunctive therapy in addition to guideline‐directed conventional treatment, showed effective improvement of symptoms and reduction of scores on the MDNS, VAS and TCSS scales.

TangBi Formula, which means a formula specializing in the treatment of limb pain or numbness caused by high blood glucose, is an empirical formula used by Academician Tong Xiaolin in the treatment of DPN. TangBi Formula's main ingredients are derived from five TCM herbs: Astragalus membranaceus (Huangqi), cassia twig (Guizhi), Ligusticum chuanxiong Hort (Chuanxiong), Radix Paeoniae Alba (Baishao), and Caulis Spatholobi (Jixueteng), whose mechanistic findings have each been found to be helpful for neuroprotection and/or blood glucose control. Astragaloside IV, a major extract of Astragalus membranaceus, has been found to be effective in the treatment of DPN by attenuating DPN myelin Schwann cell damage and improving neurological function [21]. In another study, Astragalus membranaceus extract was shown to modulate local inflammatory conditions, promote peripheral nerve regeneration (significant improvement in electrophysiological function of neurons) and potentially increase recovery from severe peripheral nerve injury in Sprague–Dawley rats [22]. An experiment related to another TCM decoction with Astragalus and cassia twig as the main ingredients proved that it can resist neuronal cell damage, reduce nociceptive sensitivity, and repair neuronal cell damage in chemotherapy‐induced neuropathic pain rats through the TNFα/IL‐1β/IL‐6/MAPK/NF‐kB pathway [17]. Ligusticum chuanxiong has a protective effect on neurons as it has been found to promote endogenous proliferation of neuronal cells and production of neural differentiation factors as well as anti‐neuroinflammation [23]. Shaoyao Gancao Tang, another agent that also contains Radix Paeoniae Alba, can exert antioxidant and anti‐inflammatory activities to inhibit neuronal apoptosis in a human tau cell model, thus showing neuroprotective effects [24]. Caulis Spatholobi, another component of TangBi Formula, may improve obesity, maintain glucose homeostasis, reduce insulin resistance, and improve hepatic steatosis through the activation of brown adipose tissue and modulation of the composition of the gut microbiota [25].

Although it has been observed in our clinical application that TangBi Formula has satisfactory efficacy on DPN patients, traditional Chinese medicine is frequently difficult to be recognized internationally due to its unique theoretical foundation, inadequate modern scientific basis and uncertain active ingredients. To our knowledge, this is the first clinical study to evaluate the safety and efficacy of TangBi Formula in DPN‐induced pain. In terms of study design, various endpoints have been used to assess the efficacy of TangBi Formula. Subjective measures including MDNS, VAS, and TCSS scales as well as objective measures such as electromyography were used in this study in order to comprehensively and accurately assess the treatment effect. Unlike neurophysiologic tests, which carry a lot of discomfort, expense, and risk of infection for patients, scoring scales are a simple, fast, and accurate screening tool for DPNs for which they are widely used in clinical practice and large‐scale clinical trials. The MDNS assesses toe sensation (including vibratory, tactile, and pinprick sensation), (including vibratory, tactile, and pinprick sensation), tendon reflexes, and distal extremity muscle strength. It is a simple, noninvasive and validated measure of distal symmetrical peripheral neuropathy that can be used in clinical practice and large clinical trials to assess distal symmetrical peripheral neuropathy [26, 27]. The TCSS is used to assess neurological function and is scored based on the patient's symptoms, signs (reflexes and sensory testing) and is also used clinically to determine if the nervous system is impaired [28]. MDNS and TCSS showed abnormal detection rates of 84.5% and 62.0%, sensitivity of 92.9% and 77.6%, specificity of 51.6% and 87.1%, and accuracy of 82.9% and 79.8%, respectively, and the combination of these two applications could improve the detection rate of DPN [29]. The VAS was used in this study to measure the intensity or frequency of pain [26] which is a common symptom of DPN. Moreover, this study used a randomized, multicenter, double‐blind method and a placebo‐controlled study design to minimize potential bias, resulting in reliable results.

From the experimental results of this study, we observed different degree of decrease in MDNS, VAS and TCSS scale scores in both groups after 24 weeks of treatment. However, the reduction in MDNS and TCSS scores was more pronounced with the addition of TangBi Formula to Methylcobalamin than with placebo. The change in MDNS and TCSS scale scores from baseline was statistically significant between the two groups after 12 and 24 weeks of treatment, and there was a significant difference in the change in VAS between the two groups after 24 weeks of treatment. Methylcobalamin remains the mainstay of treatment for PDN with the effect of promoting the regeneration of peripheral nerves, improving clinical symptoms such as neuropathic pain and neurophysiological parameters such as NCV in patients with DPN. The trophic neurologic effect of Methylcobalamin observed in this study is consistent with results reported in the literature, as the control group in this study also showed favorable results, especially in terms of changes in VAS and TCSS [30]. In the NCV results, the differences between both groups before and after treatment were subtle and not statistically significant between the two groups. In the NCV results, the differences between both groups before and after treatment were subtle and not statistically significant between the two groups. Improvement or restoration of nerve conduction generally lags far behind clinical improvement, and it was difficult to observe an effect on neurologic function with the limited time available in this study. In addition, small nerve fibers are the earliest nerve fibers to be damaged in DPN, but electrophysiological testing focuses only on large nerve fibers rather than assessing small nerve fibers [31], whereas the MDNS allows assessment of their function. The reduction of MDNS scores in the intervention group illustrated the improvement of Tangbi Formula for patients' lower extremity end sensation and distal extremity function, which is a good enhancement of sensory function and quality of life for DPN patients. Overall, the results of this study suggest that combining TangBi Formula with Methylcobalamin can effectively improve symptoms in patients with DPN, but has no clear effect on improving neurological function.

## 5 Limitations

In interpreting our findings, some limitations of this study are necessary to be recognized. Firstly, TangBi Formula is a herbal formulation, whose active ingredients and mechanism of action remain to be determined, and relevant basic research still needs to be improved. Second, because there are no definite and accurate measures of DPN, so the diagnosis relies on a combination of history, symptoms, signs, and confirmatory tests. As a result, there are no objective confirmatory evaluation criteria in clinical trials, making it difficult to compare the results of different studies. NCV provides the most sensitive and specific method for detecting DPN and is an objective measure for assessing neurologic function, but not operationally convenient. MDNS, which consists of a 15‐item self‐administered questionnaire and examination and evaluation of lower extremity vibratory sensation and ankle reflexes, is a simple, noninvasive, and validated method of evaluating the DSPN in patients with diabetes mellitus [26]. So, the MDNS scale was used as the primary outcome in this study, as well as the VAS and TCSS, which, although noninvasive and safe, are inevitably adulterated with subjective components. In subsequent studies, more objective and advanced methods should be used to assess subtle nerve functions, such as corneal confocal microscopy to detect early nerve fiber repair. Thirdly, there was a larger number of lost visits because of the overlapping of the period during which this study was conducted with the COVID‐19 outbreak. Fourth, due to this trial was conducted in mainland China and all subjects were Chinese, its generativity and efficacy need to be further evaluated in other populations.

## 6 Conclusion

In summary, this is the first randomized controlled trial to examine the efficacy and safety of Tangbi Formula in patients with DSPN. The study found that the combination of Tangbi Formula was more effective than Methylcobalamin alone in improving peripheral nerve abnormalities in patients and was well tolerated by patients without serious side effects. Tangbi Formula can be used in combination with primary therapy to improve sensory function and quality of life in patients with DPN. Improvements in sensory function are significant for diabetic patients, reducing foot ulcers in DPN and possibly enhancing proprioception and reducing the risk of falls.

## Author Contributions

F.L., Q.N., and X.T. designed the protocols. L.D., Z.F., Z.Z., X.Z., H.G., and T.G. were responsible for patient enrollment and follow‐up. X.Z., X.A., and Y.C. were responsible for organizing the data and producing the graphs. X.Z. and X.A. researched the data, participated in discussions and wrote the manuscript. All authors approved the final version of the manuscript.

## Ethics Statement

This clinical trial was approved by the Ethics Committee of Guang'anmen Hospital, Chinese Academy of Chinese Medical Sciences (No. 2016‐093‐KY‐01).

## Conflicts of Interest

The authors declare no conflicts of interest.

## Supporting information


Data S1.

## Data Availability

The datasets presented in this paper are subject to national data protection laws and ethics committee restrictions to ensure the privacy of study participants. Following publication of the main findings of the project, all data requests will be reviewed and anonymized by the trial steering committee before being submitted to the local ethics committee for approval. If it is reasonable to do so, we will provide access to anonymized data and relevant documentation to users in accordance with the data protection agreement.
