# Supplementary material for: TangBi Formula for Painful Diabetic Distal Symmetric Polyneuropathy: A Multicenter, Randomized, Double‐Blind, Placebo‐Controlled and Parallel‐Group Trial
Source: J Diabetes. 2025 Jan 5;17(1):e70045. doi: 10.1111/1753-0407.70045 (PMC11702395; doi:10.1111/1753-0407.70045)
Supplement: Supplementary file 1 — Data S1. [file JDB-17-e70045-s001.docx]

Supplementary Materials

Methods：

1. Participating investigators list

The study was a multicenter study with the centers and their directors listed in order below.

| Study center | Local investigator |
| --- | --- |
| Guang’anmen Hospital, China Academy of Chinese Medical Sciences | Liu Dong |
| the First Affiliated Hospital of Anhui University of Traditional Chinese Medicine | Zhao-hui Fang |
| Zhengzhou Hospital of Traditional Chinese Medicine | Zhong-hua Zheng |
| Hubei Provincial Hospital of Traditional Chinese Medicine | Xin-he Zuo |
| Hebei Yiling Hospital | Huai-lin Gao |
| the First Affiliated Hospital of Liaoning University of Traditional Chinese Medicine, Shenyang, Liaoning Province | Tian-shu Gao |

Supplementary Table 1: List of participating institutions and investigators

1. Study objectives

This clinical trial is designed as a multicenter, randomized, double-blind, placebo-controlled, parallel-group project. The purpose of this study was to test the hypothesis that sugar paralysis formula combined with conventional treatment is superior to conventional treatment alone in relieving clinical symptoms of DSPN and improving nerve conduction velocity in patients with DSPN. The treatment duration is set for 6 months.

1. Inclusion criteria

At screening, participants were aged 30-70 years and had a documented history of type 2 diabetes. Diagnostic criteria for type 2 diabetes included fasting plasma glucose ≥ 126 mg/dl (7.0 mmol/L), or two-hour plasma glucose ≥ 200 mg/dl (11.1 mmol/L) on an oral glucose tolerance test, or HbA1c ≥ 6.5% (48 mmol/mol), or the patient has typical symptoms of hyperglycemia or hyperglycemic crisis with random plasma glucose ≥ 200 mg/dl (11.1 mmol/L). Based on symptoms and EMG findings, participants were required to meet the diagnostic criteria for diabetic distal symmetrical polyneuropathy. The main symptoms of DSPN included numbness of the extremities; spontaneous pain (e.g., burning, tingling, dull ache); sensory abnormalities, usually glove- or sock-like, starting in the lower extremities and symmetrical; and decreased pain and temperature sensation in the feet, with reduced or absent vibration, but with basically intact motor function. The nerve conduction velocity (NCV) of any of the following 16 nerves, bilateral motor (median, ulnar, common peroneal, and superficial peroneal) and sensory (median, ulnar, common peroneal, and tibial), should be significantly lower than that of a healthy individual in the electromyogram (EMG) findings.

1. Exclusion criteria

Candidates were excluded if they had: ①Neuropathy caused by recent use of antioxidant drugs, acute infections, hepatic and renal insufficiency, acute complications of diabetes mellitus, severe cardiovascular and cerebral vascular diseases, long-term alcohol consumption and other factors. ② Combined with cardiovascular, hepatic, renal and hematopoietic system and other serious primary diseases, serum aminotransferases greater than twice the normal value, serum creatinine greater than the upper limit of normal value, psychiatric patients. ③Pregnant, preparing for pregnancy or nursing women, or those with a history of drug allergy. ④Participants in clinical studies of other drugs within the last month. ⑤ Systolic blood pressure > 160 mmHg or diastolic blood pressure > 100 mmHg. ⑥ Those who have diabetic ketoacidosis, ketoacidosis, and severe infections within the past 1 month. ⑦Alcohol and/or psychoactive substance abuse, drug abusers and dependents in the past 5 years. ⑧Other pathologies or conditions that, in the judgment of the investigator, reduce the likelihood of enrollment or complicate enrollment, such as frequent changes in the work environment, unstable living conditions, and other conditions that are likely to result in loss of visits.

1. Conditions and procedures for subject withdrawal from the study

Subjects enrolled in the study were removed from the study by the investigator after the following occurred during the course of the trial, including the occurrence of certain comorbidities, complications or specific physiologic changes that made continuation of the study inappropriate, poor subject compliance (use of medication at less than 80% of the prescribed amount or more than 120% of the prescribed amount), breaking or emergency unblinding, use of a prohibited medication as specified in the protocol. Subjects have the right to withdraw from the study at any time as described in the informed consent form. Subjects who have not formally withdrawn, but have interrupted medication and testing or cannot be contacted, are also considered to have withdrawn. The investigator made every effort to find out and document the reason for the subject's withdrawal, including whether the treatment was clearly ineffective, whether it was difficult to tolerate due to adverse effects, or whether the patient was unable to continue to participate in the clinical study due to financial or personal factors, etc. Regardless of the reason for withdrawal, a case record was maintained, the results of the last test were brought into the final analysis, and the complete dataset was analyzed to assess the efficacy of the treatment and adverse effects.

1. Ethical issues

This study has received approval from the ethics committee of Guang'anmen Hospital, China Academy of Chinese Medical Sciences (number 2016-096-KY-01). Each participating center has obtained approval from its respective local institutional review board. Prior to participating in the study, all enrolled participants will be required to provide written informed consent.

1. Bias analysis

This study was evaluated on clinical symptoms, which were influenced by a variety of factors, including blood glucose levels, current therapeutic measures, and EMG measurement conditions. This study used a randomized double-blind study design to ensure consistency of blood glucose levels between the two study groups. The administration of Mecobalamin reduced the psychological impact on the patients and improved their compliance. The doctors specialized in electromyography were also trained to fix the conditions of electromyography measurement in a uniform standard.

1. Comparison of factors affecting test evaluation

In analyzing the concurrent use of other medications during the study period, the number and percentage of cases were listed and compared. To assess medication adherence, the quantity and proportion of non-adherence (taking less than 80% or more than 120% of the prescribed dose) and adherence (taking 80-120% of the prescribed dose) were compared. The duration of dosing was calculated by subtracting the duration of the final dose from the duration of the initial dose plus one day, and the associated number of cases, mean, standard deviation, median, maximum and minimum values were calculated. The chi-square test and Fisher's exact probability method were used for all three of these items for between-group comparisons.

Results

1. Baseline data for PPS population

| Supplementary Table 2 Baseline characteristics of study patients. (PPS population) | | | | |
| --- | --- | --- | --- | --- |
| characteristic | TangBi Formula (n=80) | Control (n=73) | All participants (n=186) | p-Value |
| Age (year), Mean (SD) | 57.4 (8.64) | 57.5 (7.98) | 57.4 (8.30) | 0.864 |
| Sex, n (%) |  |  |  |  |
| Male | 49 (61.3%) | 48 (65.8%) | 97 (63.4%) |  |
| Female | 31 (38.8%) | 25 (34.2%) | 56 (36.6%) | 0.564 |
| Height (cm), Mean (SD) | 166.3 (8.50) | 168.6 (7.98) | 167.4 (8.31) | 0.151 |
| Weight (kg), Mean (SD) | 68.22 (11.241) | 71.88 (12.017) | 69.96 (11.724) | 0.069 |
| BMI (kg/m2), Mean (SD) | 24.610 (3.3025) | 25.218 (3.2999) | 24.900 (3.3045) | 0.257 |
| Duration of diabetes (year), Mean (SD) | 11.0 (6.84) | 11.3 (6.36) |  | 0.663 |
| Duration of DSPN (year), Mean (SD) | 2.554 (2.8218) | 2.345 (2.2774) |  | 0.965 |
| Comorbid with other diabetic complications (yes), n (%) | 23 (28.8%) | 15 (20.5%) |  | 0.241 |
| Past DSPN treatment history (yes), n (%) | 19 (23.8%) | 20 (27.4%) |  | 0.605 |
| Past usage of drugs for DSPN (yes), n (%) | 18 (22.5%) | 18 (24.7%) |  | 0.753 |
| Past medical history and the usage of drugs (yes), n (%) | 72 (90.0%) | 67 (91.8%) |  | 0.703 |
| Drug combination during the study period (yes), n (%) | 20 (25.0%) | 50 (68.5%) |  | 0.371 |
| Urine pregnancy test (positive), n (%) | 0 (0.0%) | 0 (0.0%) |  | 0.869 |
| FBG (mmol/L), median(p25 ~ p75) | 7.270 (6.195, 9.395) | 8.200 (7.000, 9.900) |  | 0.078 |
| HbA1c (%), median(p25 ~ p75) | 7.550 (6.750, 8.500) | 7.400 (6.600, 8.700) |  | 0.624 |

1. Primary outcomes for the PPS population

| Supplementary Table 3 Analysis of changes in primary outcomes (PPS population), Mean (SD) | | | |
| --- | --- | --- | --- |
|  | TangBi Formula (n=80) | Control (n=73) | p-Value |
| At baseline | 8.2 (8.16) | 8.3 (8.58) | 0.536 |
| 12 weeks | 5.2 (5.28) | 6.7 (6.25) | 0.057 |
| 12-week changes | -3.0 (4.29) | -1.5 (3.63) | 0.001 |
| 24 weeks | 3.0 (3.62) | 5.5 (4.20) | <0.001 |
| 24 weeks changes | -5.2 (6.04) | -2.7 (5.89) | <0.001 |

1. Qualitative analysis of the Primary outcomes

| Supplementary Table 4 Qualitative analysis of MDNS scores (FAS population), N (%) | | | | | | | | |
| --- | --- | --- | --- | --- | --- | --- | --- | --- |
|  |  |  | Left side | | | Right side | | |
|  |  |  | TangBi Formula (n=93) | Control (n=93) | p-Value | TangBi Formula (n=93) | Control (n=93) | p-Value |
| vibration sensation of thumb | At baseline | 0 : Normal | 26 (28.0%) | 40 (43.0%) | 0.095[C] | 28 (30.1%) | 40 (43.0%) | 0.144[C] |
|  |  | 1 : Decreased | 54 (58.1%) | 44 (47.3%) |  | 56 (60.2%) | 43 (46.2%) |  |
|  |  | 2 : Disappear | 13 (14.0%) | 9 (9.7%) |  | 9 (9.7%) | 10 (10.8%) |  |
|  | 24 weeks | 0 : Normal | 62 (66.7%) | 54 (58.1%) | 0.226[C] | 57 (61.3%) | 50 (53.8%) | 0.294[F] |
|  |  | 1 : Decreased | 31 (33.3%) | 39 (41.9%) |  | 36 (38.7%) | 41 (44.1%) |  |
|  |  | 2 : Disappear | 0 (0.0%) | 0 (0.0%) |  | 0 (0.0%) | 2 (2.2%) |  |
| Semmes-Weinstein Monofilament Examination of thumb | At baseline | 0 : Normal | 36 (38.7%) | 46 (49.5%) | 0.181^[C]^ | 37 (39.8%) | 40 (43.0%) | 0.899[C] |
|  |  | 1 : Decreased | 48 (51.6%) | 43 (46.2%) |  | 48 (51.6%) | 45 (48.4%) |  |
|  |  | 2 : Disappear | 9 (9.7%) | 4 (4.3%) |  | 8 (8.6%) | 8 (8.6%) |  |
|  | 24 weeks | 0 : Normal | 60 (64.5%) | 64 (68.8%) | 0.641^[F]^ | 56 (60.2%) | 55 (59.1%) | 0.612[F] |
|  |  | 1 : Decreased | 32 (34.4%) | 29 (31.2%) |  | 37 (39.8%) | 36 (38.7%) |  |
|  |  | 2 : Disappear | 1 (1.1%) | 0 (0.0%) |  | 0 (0.0%) | 2 (2.2%) |  |
| Needling sensation on dorsal side of thumb | At baseline | 0 : Normal | 78 (83.9%) | 75 (80.6%) | 0.565^[C]^ | 76 (81.7%) | 79 (84.9%) | 0.555[C] |
|  |  | 2 : Disappear | 15 (16.1%) | 18 (19.4%) |  | 17 (18.3%) | 14 (15.1%) |  |
|  | 24 weeks | 0 : Normal | 86 (92.5%) | 81 (87.1%) | 0.226^[C]^ | 87 (93.5%) | 86 (92.5%) | 0.774[C] |
|  |  | 2 : Disappear | 7 (7.5%) | 12 (12.9%) |  | 6 (6.5%) | 7 (7.5%) |  |
| Finger extensor muscle strength | At baseline | 0 : Normal | 65 (69.9%) | 57 (61.3%) | 0.531^[F]^ | 59 (63.4%) | 61 (65.6%) | 0.645[F] |
|  |  | 1 : Slight to moderate weakness | 17 (18.3%) | 27 (29.0%) |  | 26 (28.0%) | 24 (25.8%) |  |
|  |  | 2 : Severe weakness | 9 (9.7%) | 8 (8.6%) |  | 6 (6.5%) | 8 (8.6%) |  |
|  |  | 3 : No movement | 2 (2.2%) | 1 (1.1%) |  | 2 (2.2%) | 0 (0.0%) |  |
|  | 24 weeks | 0 : Normal | 73 (78.5%) | 68 (73.1%) | 0.382[F] | 74 (79.6%) | 64 (68.8%) | 0.180[F] |
|  |  | 1 : Slight to moderate weakness | 20 (21.5%) | 23 (24.7%) |  | 18 (19.4%) | 28 (30.1%) |  |
|  |  | 2 : Severe weakness | 0 (0.0%) | 2 (2.2%) |  | 1 (1.1%) | 1 (1.1%) |  |
|  |  | 3 : No movement | 0 (0.0%) | 0 (0.0%) |  | 0 (0.0%) | 0 (0.0%) |  |
| Thumb extensor muscle strength | At baseline | 0 : Normal | 82 (88.2%) | 71 (76.3%) | 0.531[F] | 66 (71.0%) | 68 (73.1%) | 0.967[F] |
|  |  | 1 : Slight to moderate weakness | 11 (11.8%) | 21 (22.6%) |  | 20 (21.5%) | 19 (20.4%) |  |
|  |  | 2 : Severe weakness | 0 (0.0%) | 1 (1.1%) |  | 6 (6.5%) | 6 (6.5%) |  |
|  |  | 3 : No movement | 0 (0.0%) | 0 (0.0%) |  | 1 (1.1%) | 0 (0.0%) |  |
|  | 24 weeks | 0 : Normal | 82 (88.2%) | 71 (76.3%) | 0.054[F] | 74 (79.6%) | 70 (75.3%) | 0.483[C] |
|  |  | 1 : Slight to moderate weakness | 11 (11.8%) | 21 (22.6%) |  | 19 (20.4%) | 23 (24.7%) |  |
|  |  | 2 : Severe weakness | 0 (0.0%) | 1 (1.1%) |  | 0 (0.0%) | 0 (0.0%) |  |
|  |  | 3 : No movement | 0 (0.0%) | 0 (0.0%) |  | 0 (0.0%) | 0 (0.0%) |  |
| Ankle dorsiflexor muscle strength | At baseline | 0 : Normal | 66 (71.0%) | 69 (74.2%) | 0.749[C] | 65 (69.9%) | 64 (68.8%) | 0.901[F] |
|  |  | 1 : Slight to moderate weakness | 20 (21.5%) | 16 (17.2%) |  | 23 (24.7%) | 23 (24.7%) |  |
|  |  | 2 : Severe weakness | 7 (7.5%) | 8 (8.6%) |  | 4 (4.3%) | 6 (6.5%) |  |
|  |  | 3 : No movement | 0 (0.0%) | 0 (0.0%) |  | 1 (1.1%) | 0 (0.0%) |  |
|  | 24 weeks | 0 : Normal | 79 (84.9%) | 65 (69.9%) | 0.022[F] | 82 (88.2%) | 66 (71.0%) | 0.005[F] |
|  |  | 1 : Slight to moderate weakness | 14 (15.1%) | 27 (29.0%) |  | 10 (10.8%) | 26 (28.0%) |  |
|  |  | 2 : Severe weakness | 0 (0.0%) | 1 (1.1%) |  | 1 (1.1%) | 1 (1.1%) |  |
|  |  | 3 : No movement | 0 (0.0%) | 0 (0.0%) |  | 0 (0.0%) | 0 (0.0%) |  |
| Biceps reflex | At baseline | 0 : Exists | 80 (86.0%) | 79 (84.9%) | 0.398^[C]^ | 85 (91.4%) | 81 (87.1%) | 0.526[F] |
|  |  | 1 : Hyperactive | 5 (5.4%) | 9 (9.7%) |  | 3 (3.2%) | 6 (6.5%) |  |
|  |  | 2 : Disappear | 8 (8.6%) | 5 (5.4%) |  | 5 (5.4%) | 6 (6.5%) |  |
|  | 24 weeks | 0 : Exists | 85 (91.4%) | 84 (90.3%) | 1.000^[F]^ | 89 (95.7%) | 86 (92.5%) | 0.456[F] |
|  |  | 1 : Hyperactive | 7 (7.5%) | 8 (8.6%) |  | 4 (4.3%) | 5 (5.4%) |  |
|  |  | 2 : Disappear | 1 (1.1%) | 1 (1.1%) |  | 0 (0.0%) | 2 (2.2%) |  |
| Triceps reflex | At baseline | 0 : Exists | 82 (88.2%) | 79 (84.9%) | 0.646^[C]^ | 86 (92.5%) | 79 (84.9%) | 0.208[F] |
|  |  | 1 : Hyperactive | 4 (4.3%) | 7 (7.5%) |  | 2 (2.2%) | 6 (6.5%) |  |
|  |  | 2 : Disappear | 7 (7.5%) | 7 (7.5%) |  | 5 (5.4%) | 8 (8.6%) |  |
|  | 24 weeks | 0 : Exists | 88 (94.6%) | 85 (91.4%) | 0.219^[F]^ | 89 (95.7%) | 85 (91.4%) | 0.495[F] |
|  |  | 1 : Hyperactive | 2 (2.2%) | 7 (7.5%) |  | 3 (3.2%) | 6 (6.5%) |  |
|  |  | 2 : Disappear | 3 (3.2%) | 1 (1.1%) |  | 1 (1.1%) | 2 (2.2%) |  |
| Quadriceps reflex | At baseline | 0 : Exists | 81 (87.1%) | 78 (83.9%) | 0.796^[C]^ | 79 (84.9%) | 79 (84.9%) | 0.931[C] |
|  |  | 1 : Hyperactive | 7 (7.5%) | 8 (8.6%) |  | 8 (8.6%) | 7 (7.5%) |  |
|  |  | 2 : Disappear | 5 (5.4%) | 7 (7.5%) |  | 6 (6.5%) | 7 (7.5%) |  |
|  | 24 weeks | 0 : Exists | 88 (94.6%) | 77 (82.8%) | 0.004[F] | 87 (93.5%) | 81 (87.1%) | 0.264[F] |
|  |  | 1 : Hyperactive | 2 (2.2%) | 14 (15.1%) |  | 4 (4.3%) | 10 (10.8%) |  |
|  |  | 2 : Disappear | 3 (3.2%) | 2 (2.2%) |  | 2 (2.2%) | 2 (2.2%) |  |
| Achilles tendon reflex | At baseline | 0 : Exists | 68 (73.1%) | 74 (79.6%) | 0.416^[C]^ | 67 (72.0%) | 72 (77.4%) | 0.700[C] |
|  |  | 1 : Hyperactive | 15 (16.1%) | 9 (9.7%) |  | 15 (16.1%) | 12 (12.9%) |  |
|  |  | 2 : Disappear | 10 (10.8%) | 10 (10.8%) |  | 11 (11.8%) | 9 (9.7%) |  |
|  | 24 weeks | 0 : Exists | 85 (91.4%) | 65 (69.9%) | <0.001^[F]^ | 86 (92.5%) | 69 (74.2%) | 0.003[F] |
|  |  | 1 : Hyperactive | 6 (6.5%) | 21 (22.6%) |  | 5 (5.4%) | 18 (19.4%) |  |
|  |  | 2 : Disappear | 2 (2.2%) | 7 (7.5%) |  | 2 (2.2%) | 6 (6.5%) |  |
| Note: [C] The P-Value was calculated using the chi-square test. [F]P-Value is calculated using Fisher's exact test. | | | | | | | | |

1. Results of the secondary outcomes

| Supplementary Table 5 Analysis of changes in NCV (FAS population), Mean (SD) | | | | |
| --- | --- | --- | --- | --- |
| Variable |  | TangBi Formula (n=93) | Control (n=93) | p-Value |
| SNCV |  |  |  |  |
| Left median nerve conduction velocity (m/s) | At baseline | 48.673 (8.3398) | 49.684 (9.2206) | 0.450 |
|  | 24 weeks | 49.548 (9.7501) | 50.739 (9.3106) | 0.385 |
|  | 24 weeks changes | 0.877 (7.1499) | 1.129 (5.1762) | 0.955 |
| Left ulnar nerve conduction velocity (m/s) | At baseline | 53.256 (8.0280) | 52.403 (8.7076) | 0.793 |
|  | 24 weeks | 51.250 (9.0422) | 52.218 (9.0748) | 0.473 |
|  | 24 weeks changes | -1.582 (7.4962) | -0.270 (5.2295) | 0.881 |
| Left sural nerve conduction velocity (m/s) | At baseline | 47.405 (10.5386) | 46.752 (9.6432) | 0.675 |
|  | 24 weeks | 47.222 (9.2647) | 46.080 (9.1077) | 0.414 |
|  | 24 weeks changes | 0.258 (8.0780) | -0.724 (5.1374) | 0.261 |
| Left superficial peroneal nerve conduction velocity (m/s) | At baseline | 40.215 (11.9248) | 39.940 (10.4494) | 0.939 |
|  | 24 weeks | 40.636 (9.3025) | 42.234 (9.5180) | 0.196 |
|  | 24 weeks changes | 0.973 (6.9510) | 2.198 (7.0177) | 0.590 |
| Right median nerve conduction velocity (m/s) | At baseline | 48.252 (8.8851) | 49.153 (7.9716) | 0.283 |
|  | 24 weeks | 48.516 (9.6455) | 49.781 (9.0049) | 0.272 |
|  | 24 weeks changes | 0.298 (7.3661) | 0.758 (6.0488) | 0.458 |
| Right ulnar nerve conduction velocity (m/s) | At baseline | 52.103 (7.8121) | 51.561 (9.2010) | 0.679 |
|  | 24 weeks | 51.531 (7.9393) | 52.446 (8.9615) | 0.473 |
|  | 24 weeks changes | -0.425 (6.9954) | 0.816 (5.0658) | 0.132 |
| Right sural nerve conduction velocity (m/s) | At baseline | 47.058 (9.3713) | 46.307 (9.1472) | 0.601 |
|  | 24 weeks | 46.955 (9.3698) | 45.435 (9.0874) | 0.279 |
|  | 24 weeks changes | 0.141 (6.3751) | -0.913 (5.3907) | 0.527 |
| Right superficial peroneal nerve conduction velocity (m/s) | At baseline | 40.832 (11.8015) | 39.984 (10.6252) | 0.642 |
|  | 24 weeks | 40.968 (9.4602) | 42.722 (9.5484) | 0.242 |
|  | 24 weeks changes | 0.605 (7.2628) | 2.754 (7.0273) | 0.125 |
| MNCV |  |  |  |  |
| Left median nerve conduction velocity (m/s) | At baseline | 52.451 (7.2724) | 52.247 (5.2521) | 0.454 |
|  | 24 weeks | 52.618 (6.9007) | 52.807 (5.3887) | 0.641 |
|  | 24 weeks changes | 0.098 (4.6577) | 0.312 (3.4282) | 0.56 |
| Left ulnar nerve conduction velocity (m/s) | At baseline | 53.706 (6.8870) | 53.481 (6.7493) | 0.93 |
|  | 24 weeks | 53.931 (6.9379) | 53.750 (6.5902) | 0.924 |
|  | 24 weeks changes | 0.156 (4.8739) | 0.347 (5.4376) | 0.703 |
| Left peroneal nerve conduction velocity (m/s) | At baseline | 43.1318 (7.33652) | 42.8643 (6.95823) | 0.911 |
|  | 24 weeks | 43.1375 (6.23477) | 43.8870 (6.81980) | 0.166 |
|  | 24 weeks changes | 0.0056 (6.47564) | 0.9517 (6.07817) | 0.078 |
| Left tibial nerve conduction velocity (m/s) | At baseline | 39.402 (6.8620) | 39.001 (6.6123) | 0.598 |
|  | 24 weeks | 39.451 (5.8108) | 39.995 (6.9666) | 0.721 |
|  | 24 weeks changes | 0.050 (4.4075) | 0.921 (3.6423) | 0.479 |
| Right median nerve conduction velocity (m/s) | At baseline | 52.212 (8.1671) | 53.754 (5.6816) | 0.58 |
|  | 24 weeks | 52.765 (7.5132) | 53.636 (5.5067) | 0.905 |
|  | 24 weeks changes | 0.553 (5.3260) | -0.025 (3.4573) | 0.464 |
| Right ulnar nerve conduction velocity (m/s) | At baseline | 54.667 (8.5810) | 54.735 (6.3335) | 0.727 |
|  | 24 weeks | 54.721 (7.9778) | 54.952 (7.7930) | 0.435 |
|  | 24 weeks changes | 0.056 (5.0598) | 0.336 (6.2178) | 0.62 |
| Right peroneal nerve conduction velocity (m/s) | At baseline | 43.1048 (7.88239) | 42.7933 (7.29918) | 0.785 |
|  | 24 weeks | 43.7444 (6.52362) | 43.5174 (7.68855) | 0.831 |
|  | 24 weeks changes | 0.6606 (5.80669) | 0.6441 (5.99922) | 0.311 |
| Right tibial nerve conduction velocity (m/s) | At baseline | 39.529 (8.1058) | 38.751 (6.6677) | 0.485 |
|  | 24 weeks | 40.051 (7.4370) | 39.681 (7.2250) | 0.736 |
|  | 24 weeks changes | 0.511 (4.6410) | 0.791 (3.7185) | 0.744 |

1. Other outcomes

| Supplementary Table 6: Analysis of changes in VAS (PPS population), Mean (SD) | | | |
| --- | --- | --- | --- |
|  | TangBi Formula (n=80) | Control (n=73) | p-Value |
| At baseline | 4.0 (3.42) | 2.9 (3.23) | 0.038 |
| 12 weeks | 2.9 (2.84) | 2.1 (2.54) | 0.082 |
| 12-week changes | -1.1 (1.32) | -0.9 (1.27) | 0.183 |
| 24 weeks | 2.0 (2.42) | 1.6 (2.25) | 0.254 |
| 24 weeks changes | -2.0 (1.99) | -1.3 (1.77) | 0.041 |

| Supplementary Table 7: Analysis of changes in TCSS (PPS population), Mean (SD) | | | |
| --- | --- | --- | --- |
|  | TangBi Formula (n=80) | Control (n=73) | p-Value |
| At baseline | 7.7 (2.83) | 7.0 (2.03) | 0.285 |
| 12 weeks | 6.0 (2.37) | 5.8 (2.09) | 0.606 |
| 12-week changes | -1.7 (1.72) | -1.3 (1.39) | 0.064 |
| 24 weeks | 4.9 (2.18) | 5.0 (2.03) | 1.000 |
| 24 weeks changes | -2.8 (2.07) | -2.1 (1.39) | 0.068 |
